# Supplementary material for: Sleep phenotyping in a rat model of susceptibility to substance use disorders
Source: PLoS One. 2025 May 29;20(5):e0324459. doi: 10.1371/journal.pone.0324459 (PMC12121824; doi:10.1371/journal.pone.0324459)
Supplement: S1 Table — Outliers identified by the Grubbs’ test were removed from the statistical analysis. (DOCX) [file pone.0324459.s001.docx]

**S1 Table. Summary of statistical results.**

| **Figure 1** | | | |
| --- | --- | --- | --- |
| **Parameter** | **Outlier Tests Grubbs’/ROUT** | | **Statistical test excluding outliers** |
| **1B** Stage duration across 24 hours | | | |
| NREM sleep W | ns/ns | | 2-way ANOVA, *stage* x *rat line* F(2, 48) = 5.793 p=0.0056.  Fisher’s LSD post-hoc test msP vs Wistar:  NREM sleep p=0.0163 *  REM sleep p=0.9  wake p=0.0247 * |
| NREM sleep msP | ns/ns | |  |
| REM W | ns/ns | |  |
| REM msP | ns/ns | |  |
| Wake W | ns/ns | |  |
| Wake msP | ns/ns | |  |
| **1C** stage duration during light and dark phase | | | |
| NREM sleep W (L) | ns/ns | | 2-way ANOVA, *stage* x *rat line* F(5, 95)=3.9 p=0.0057.  Fisher’s LSD post-hoc test msP vs Wistar:  NREM (L) p=0.40  Wake (L) p=0.31  REM (L) p=0.85  NREM (D) p=0.046 *  Wake (D) p=0.0013 **  REM (D) p=0.38 |
| NREM sleep msP (L) | ns/ns | |  |
| REM sleep W (L) | ns/ns | |  |
| REM sleep msP (L) | ns/ns | |  |
| Wake W (L) | ns/ns | |  |
| Wake msP (L) | ns/ns | |  |
| NREM sleep W (D) | ns/ns | |  |
| NREM sleep msP (D) | 1/1 | |  |
| REM sleep W (D) | ns/ns | |  |
| REM sleep msP (D) | ns/ns | |  |
| Wake W (D) | ns/ns | |  |
| Wake msP (D) | ns/ns | |  |
| **1D** stage duration 2-hour bin | | | |
| Wake W | ns | | Mixed effects Model (REML):  *time* x *rat* *line* F(11, 190) = 2.63, p=0.038,  *rat line* F(1, 190) = 1.34 p=0.25 |
| Wake msP | 2 (in different time points)/2 | |  |
| NREM sleep W | ns/ns | | Mixed effects Model (REML):  *time* x *rat line* F(11, 191) = 1.63, p=0.09,  *rat line* F(1, 191) = 3.89, p=0.05 |
| NREM sleep msP | 1/ns | |  |
| REM sleep W | ns/ns | | Mixed effects Model (REML):  *time* x *rat* *line* F(11, 175) = 3 p=0.0011,  *rat line* F(1,16)=0.006, p=0.93 |
| REM sleep msP | 1/ns | |  |
| **1E** number of episodes during light and dark phase | | | |
| NREM sleep W (L) | ns/ns | | 2-way ANOVA,  *state* x *rat line* F(5, 90)=1.13, p=0.35  *rat line* F(1, 90) = 4.68 p=0.033 |
| NREM sleep msP (L) | ns/ns | |  |
| REM sleep W (L) | ns/ns | |  |
| REM sleep msP (L) | ns/ns | |  |
| Wake W (L) | ns/ns | |  |
| Wake msP (L) | ns/ns | |  |
| NREM sleep W (D) | ns/ns | |  |
| NREM sleep msP (D) | ns/ns | |  |
| REM sleep W (D) | ns/ns | |  |
| REM sleep msP (D) | ns/ns | |  |
| Wake W (D) | ns/ns | |  |
| Wake msP (D) | ns/ns | |  |
| **1F.** number of sleep attempts in 24hr | | | |
| W | ns/ns | | Mann Whitney p=0.0468 |
| msP | ns/ns | |  |
| **1G.** sleep fragmentation index | | | |
| W | ns/ns | | Unpaired t-test p=0.036 |
| msP | ns/ns | |  |
| **1H.** Number of transitions | | | |
| NREM-REM W | ns/ns | | Mixed effect model (REML),  *transition* x *rat line* F(5,92)=1.16, p=0.33  *rat line* F(1,92) = 3.67 p=0.058  transition F(1.403,25082)=274 p<0.0001  (# excluding 2 outliers, rat line F(1,91)=3.72 p=0.057) |
| NREM-REM msP | 1/1 | |  |
| NREM-Wake W | ns/ns | |  |
| NREM-Wake msP | ns/ns | |  |
| Wake-REM W | 1/2 # | |  |
| Wake-REM msP | ns/ns | |  |
| Wake-NREM W | 1 /1 | |  |
| Wake-NREM msP | ns/ns | |  |
| REM-NREM W | ns/ns | |  |
| REM-NREM msP | ns/ns | |  |
| REM-Wake W | 1/1 | |  |
| REM-Wake msP | ns/ns | |  |
| **1I.** % of behavior | | | |
| Active Wake Light phase Wistar | ns/ns | | Mixed effect model (REML),  *state* x *rat line* F(3, 62)=6.76 p=0.0005, *state* F(1.094, 22.61)=77.56, p<0.0001,  *rat line* F(1,62)=3.082E-017 p>0.99 |
| Active Wake Light phase msP | ns/ns | |  |
| Quiet Wake Light phase Wistar | ns/ns | |  |
| Quiet Wake Light phase msP | ns/ns | |  |
| Active Wake Dark phase Wistar | 1 /1 | |  |
| Active Wake Dark phase msP | ns/ns | |  |
| Quiet Wake Dark phase Wistar | 1/1 | |  |
| Quiet Wake Dark phase msP | ns/ns | |  |
| **Šídák's multiple comparisons test** | | | **Adjusted p** |
| Active Wake Light phase | | | |
| Wistar vs msP | | | 0,032 * |
| Quiet Wake Light phase | | | |
| Wistar vs msP | | | 0,032 * |
| Active Wake Dark phase | | | |
| Wistar vs msP | | | 0.87 |
| Quiet Wake Dark phase | | | |
| Wistar vs msP | | | 0.87 |
| Wistar | | | |
| Active Wake L vs. Quiet Wake L | | | 0,64 |
| Active Wake L vs. Active Wake D | | | 0,053 |
| Active Wake L vs. Quiet Wake D | | | <0.0006 *** |
| Quiet Wake L vs. Active Wake D | | | <0.0006 *** |
| Quiet Wake L vs. Quiet Wake D | | | 0.053 |
| Active Wake D vs. Quiet Wake D | | | <0,0001 **** |
| msP | | | |
| Active Wake L vs. Quiet Wake L | | | 0,5 |
| Active Wake L vs. Active Wake D | | | <0,0001 **** |
| Active Wake L vs. Quiet Wake D | | | 0,032 * |
| Quiet Wake L vs. Active Wake D | | | 0,032 * |
| Quiet Wake L vs. Quiet Wake D | | | <0,0001 **** |
| Active Wake D vs. Quiet Wake D | | | <0,0001 **** |
| **Figure 2** | | | |
| **Parameter** | | **Outlier Tests Grubbs’/ ROUT** | **Statistical test excluding outliers** |
| slow wave activity | | ns/ns | Unpaired t-test p=0.26 |
| Sigma power | | ns/ns | Unpaired t-test p=0.0001**** |
| Beta power during NREM sleep | | ns/ns | Unpaired t-test p=0.0001**** |
| REM low theta power | | ns/ns | Unpaired t-test p=0.0785 |
| REM high theta power | | ns/ns | Mann-Whitney p=0.9 |
| REM beta power | | ns/ns | Unpaired t-test p=0.0009*** |
| Wake high theta | | ns/ns | Unpaired t-test p=0.0116* |
| Wake beta power | | ns/ns | Unpaired t-test p=0.0011** |
| Wake theta/beta power | | ns/ns | Unpaired t-test p=0.0082** |
| **Figure 3** | | | |
| **Parameter** | | **Outlier Test Grubbs’/ ROUT** | **Statistical test excluding outliers** |
| slow wave activity | | ns/ns | Unpaired t-test p=0.37 |
| Sigma power | | ns/ns | Unpaired t-test p=0.0001 *** |
| Beta power during NREM sleep | | ns/ns | Unpaired t-test p<0.0001**** |
| REM sleep low theta power | | ns/ns | Mann Whitney p=0.1359 |
| REM sleep high theta power | | ns/ns | Unpaired t-test p=0.52 |
| REM sleep beta power | | ns/ns | Unpaired t-test p=0.0036** |
| Wake high theta power | | ns/ns | Mann Whitney p=0.32 |
| Wake beta power | | ns/ns | Unpaired t-test p=0.0014** |
| Wake theta/beta power | | ns/1 | Mann Whitney p=0.0037** (p=0.0079 excluding 1 outlier) |
| **Figure 4** | | | |
| **Parameter** | | **Outlier Test Grubbs’/ROUT** | **Statistical test excluding outliers** |
| **4A.** SWA | | | |
| Wistar | | 8 (in different time points)/4 | Mixed effects model (REML),  *time* x *rat line* F(23,341)=3.92, p<0.0001****  *time* F(5.27, 78.1)=12.9, p<0.0001****  *rat line* F(1,16)=1.13, p=0.3 |
| msP | | 5/2 |  |
| **4B.** Light period SWA change | | | |
| Wistar | | ns/ns | Unpaired t test p=0.0042** |
| msP | | 1/1 |  |
| **4C.** Dark period SWA change | | | |
| Wistar | | ns/ns | Unpaired t-test p=0.0154* |
| msP | | ns/ns |  |
| **4E.** SW amplitude | | | |
| Wistar Early sleep | | ns/ns | Mixed effects model (REML),  *time* x *rat line* F(1, 15)=4.87, p=0.043, *time* F(1, 15)=127.6, p<0.0001,  *rat line* F(1,16)=4.3, p=0.055 |
| msP Early sleep | | ns/ns |  |
| Wistar Late Sleep | | 1/ns |  |
| msP Late sleep | | ns/ns |  |
| **Šídák's multiple comparisons test** | | | **Adjusted p** |
| Early sleep | | | |
| Wistar vs msP | | | 0.019* |
| Late sleep | | | |
| Wistar vs msP | | | 0.49 |
| Wistar | | | |
| Early vs Late Sleep | | | <0.0001 **** |
| msP | | | |
| Early vs Late Sleep | | | <0.0001 **** |
| **4F.** SW slope | | | |
| Wistar Early sleep | | ns/ns | Mixed effects model (REML),  *time* x *rat line* F(1, 15)=6.27, p=0.024, *time* F(1, 15)=126.7, p<0.0001,  *rat line* F(1,16)=4.73, p=0.045 |
| msP Early sleep | | ns/ns |  |
| Wistar Late Sleep | | 1/ns |  |
| msP Late sleep | | ns/ns |  |
| **Šídák's multiple comparisons test** | | | **Adjusted p** |
| Early Sleep | | | |
| Wistar vs msP | | | 0.011 * |
| Late sleep | | | |
| Wistar vs msP | | | 0.53 |
| Wistar | | | |
| Early vs Late Sleep | | | <0.0001**** |
| msP | | | |
| Early vs Late Sleep | | | <0.0001**** |
| **4G.** SW Duration | | | |
| Wistar Early sleep | | ns/ns | Mixed effects model (REML),  *time* x *rat line* F(1, 15)=6.369, p=0.0234, *time* F(1, 15)=6.129, p=0.026,  *rat line* F(1,16)=5.386, p=0.034 |
| msP Early sleep | | 1/ns |  |
| Wistar Late Sleep | | ns/ns |  |
| msP Late sleep | | ns/ns |  |
| **Šídák's multiple comparisons test** | | | **Adjusted p** |
| Early sleep | | | |
| Wistar vs msP | | | 0.9 |
| Late sleep | | | |
| Wistar vs msP | | | 0.0046** |
| Wistar | | | |
| Early vs Late Sleep | | | 0.0051 ** |
| msP | | | |
| Early vs Late Sleep | | | 0.55 |
| **4H.** SW frequency | | | |
| Wistar Early sleep | | ns/ns | Mixed effects model (REML),  *time* x *rat line* F(1, 15)=4.99, p=0.041, *time* F(1, 15)=3.56, p=0.078,  *rat line* F(1,16)=2.55, p=0.13 |
| msP Early sleep | | 1/ns |  |
| Wistar Late Sleep | | ns/ns |  |
| msP Late sleep | | ns/ns |  |
| **Šídák's multiple comparisons test** | | | **Adjusted p** |
| Early Sleep | | | |
| Wistar vs msP | | | 0.95 |
| Late sleep | | | |
| Wistar vs msP | | | 0.034* |
| Wistar | | | |
| Early vs Late Sleep | | | 0.0185* |
| msP | | | |
| Early vs Late Sleep | | | 0.96 |
| **4I.** SW Incidence | | | |
| Wistar Early sleep | | ns/ns | 2-way repeated measure ANOVA,  *time* x *rat line* F(1, 16)=13.55, p=0.002, *time* F(1, 16)=28.57, p<0.0001,  *rat line* F(1,16)=0.235, p=0.634 |
| msP Early sleep | | ns/ns |  |
| Wistar Late Sleep | | ns/ns |  |
| msP Late sleep | | ns/ns |  |
| **Šídák's multiple comparisons test** | | | **Adjusted p** |
| Early Sleep | | | |
| Wistar vs msP | | | 0.57 |
| Late sleep | | | |
| Wistar vs msP | | | 0.14 |
| Wistar | | | |
| Early vs Late Sleep | | | <0.0001 **** |
| msP | | | |
| Early vs Late Sleep | | | 0.45 |
| **Figure 5** | | | |
| **Parameter** | | **Outlier Test Grubbs’/ROUT** | **Statistical test excluding outliers** |
| **5A.** NREM sigma power | | | |
| Wistar | | 3 (in different time points)/1 | Mixed effect model (REML)  *time* x *rat line* F(23,347)=3.32, p<0.0001****  *time* F(4.14, 62.41)=20.83, p<0.0001****  *rat line* F(1, 16)=30.97, p<0.0001****  Šídák's multiple comparisons test |
| msP | | 4/ns |  |
| **5B.** Light period sigma change | | | |
| Wistar | | 1/ns | Unpaired t-test p=0.021 |
| msP | | ns/ns |  |
| **5D.** Spindle Amplitude | | | |
| Wistar Early sleep | | ns/ns | Mixed effect model (REML)  *time* x *rat line* F(1, 15)=1.385, p=0.46, *time* F(1, 15)=3.66, p=0.07,  *rat line* F(1,16)=45.15, p<0.0001 |
| msP Early sleep | | 1/ns |  |
| Wistar Late Sleep | | ns/ns |  |
| msP Late sleep | | ns/ns |  |
| **5E.** Spindle Incidence | | | |
| Wistar Early sleep | | ns/ns | 2-way repeated measure ANOVA,  *time* x *rat line* F(1, 16)=26.09, p=0.0001, *time* F(1, 16)=124.8, p<0.0001,  *rat line* F(1,16)=23.68, p=0.0002 |
| msP Early sleep | | ns/ns |  |
| Wistar Late Sleep | | ns/ns |  |
| msP Late sleep | | ns/ns |  |
| **Šídák's multiple comparisons test** | | | **Adjusted p** |
| Early Sleep | | | |
| Wistar vs msP | | | 0.0142 * |
| Late sleep | | | |
| Wistar vs msP | | | <0.0001 **** |
| Wistar | | | |
| Early vs Late Sleep | | | <0.0001 **** |
| msP | | | |
| Early vs Late Sleep | | | 0.0011 ** |
| **5F.** Spindle duration | | | |
| Wistar Early sleep | | ns/ns | Mixed effect model (REML)  *Time x rat line* F(1,15)=2.91 p=0.1  *Time* (F1,15)=15.08 p=0.0015  *rat line* F(1,16)=22.52 p=0.0002 |
| msP Early sleep | | ns/ns |  |
| Wistar Late Sleep | | ns/ns |  |
| msP Late sleep | | 1/ns |  |
| **G.** Spindle Frequency | | | |
| Wistar Early sleep | | ns/ns | 2-way repeated measure ANOVA,  *time* x *rat line* F(1, 16)=2.742, p=0.117, *time* F(1, 16)=0.29, p=0.597,  *rat line* F(1,16)=15.69, p=0.001 |
| msP Early sleep | | ns/ns |  |
| Wistar Late Sleep | | ns/ns |  |
| msP Late sleep | | ns/ns |  |
| **H.** Oscillations per spindle | | | |
| Wistar Early sleep | | ns/ns | 2-way repeated measure ANOVA,  *time* x *rat line* F(1, 16)=0.971, p=0.339, *time* F(1, 16)=8.273, p=0.011,  *rat line* F(1,16)=25.81, p=0.001 |
| msP Early sleep | | ns/ns |  |
| Wistar Late Sleep | | ns/ns |  |
| msP Late sleep | | ns/ns |  |
