# Supplementary figures and images for: Sleep phenotyping in a rat model of susceptibility to substance use disorders
Source: PLoS One. 2025 May 29;20(5):e0324459. doi: 10.1371/journal.pone.0324459 (PMC12121824; doi:10.1371/journal.pone.0324459)

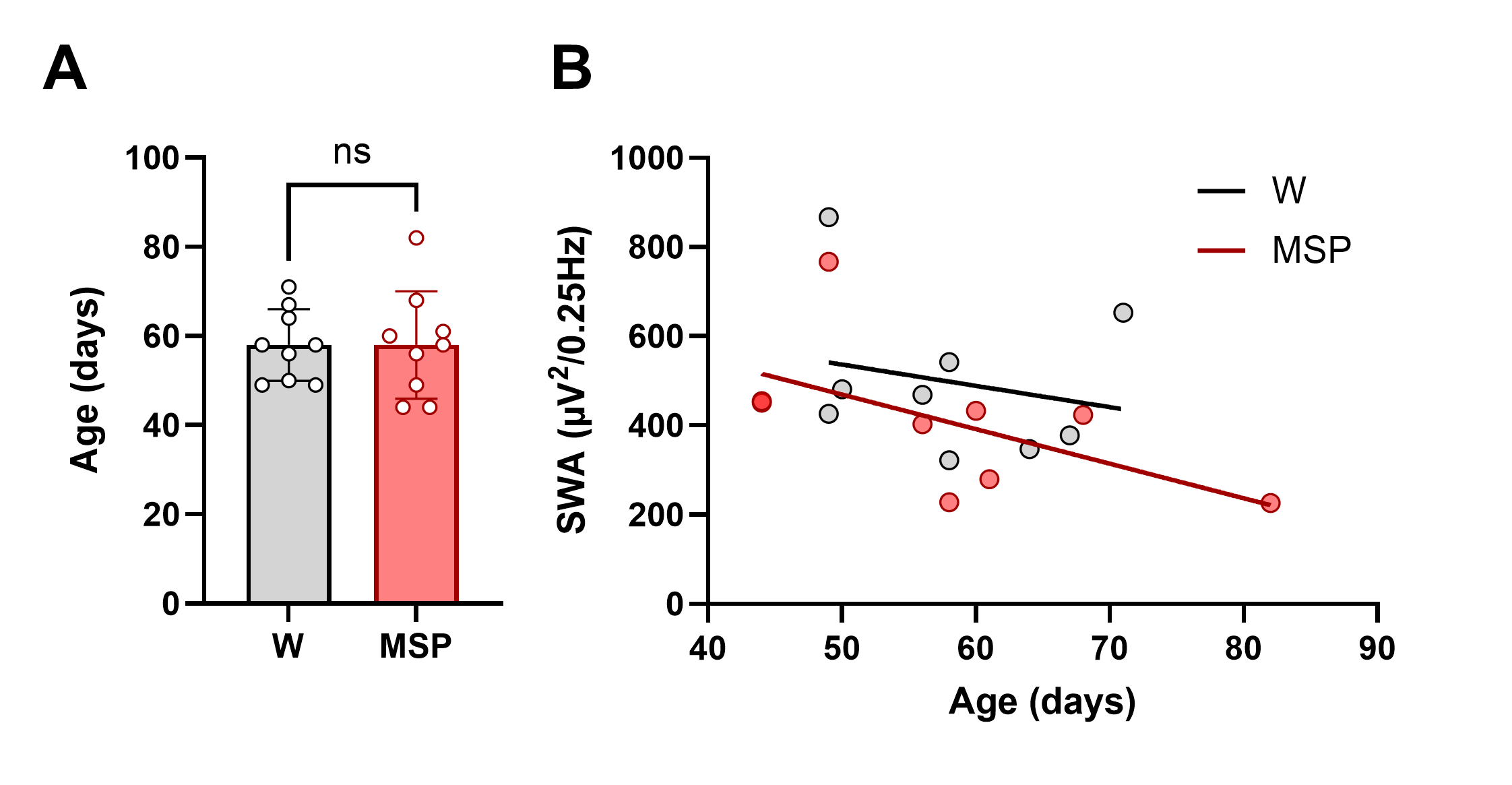

Supplement: S1 Fig — A) age at baseline recordings for each individual rat. W = Wistars, 58 ± 8 days, msP 58 ± 12 days, unpaired t-test p > 0.99. B) mean NREM SWA levels (24 hours) across age from frontal cortex. Each symbol refers to a single animal. (TIF) [file pone.0324459.s002.tif]

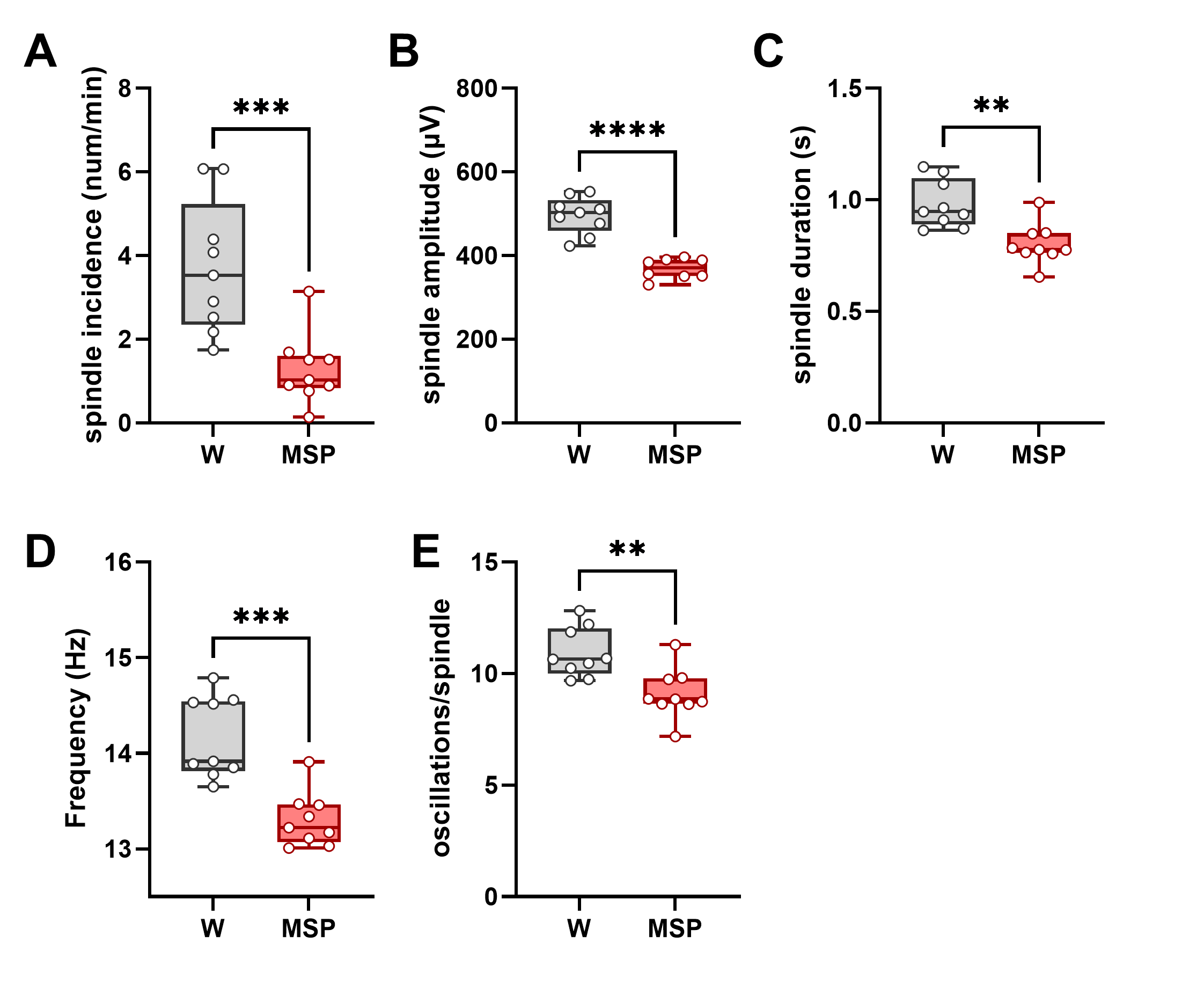

Supplement: S2 Fig — A) spindle incidence B) spindle amplitude. C) spindle duration. D) spindle frequency. E) number of oscillations per spindle. W = Wistar, unpaired t-test. ** p < 0.01, ***p < 0.001. (TIF) [file pone.0324459.s003.tif]
