# Supplementary material for: Sleep phenotyping in a rat model of susceptibility to substance use disorders
Source: PLoS One. 2025 May 29;20(5):e0324459. doi: 10.1371/journal.pone.0324459 (PMC12121824; doi:10.1371/journal.pone.0324459)
Supplement: Attachment — Submitted filename: Response_to_Reviewers.docx [file pone.0324459.s005.docx]

**Reviewer #1:** Major points:

1.1 Discussion could benefit from interpretation of the findings in relation to propensity for alcohol taking (e.g., how does potential effect on cognition, or learning and memory, impact alcohol drinking, or propensity to start drinking).

We thank the reviewer for the opportunity to discuss further these important points. We expanded our discussion on this part as follows (page 19):

*“[...] These and our findings suggest that the neurobiological alterations underlying deficits in sleep slow waves and spindles, and possibly in learning, found in msP rats and in youngsters at risk of AUD might constitute an endophenotype which contributes to their propensity for alcohol use. The relationship between cognitive functions and vulnerability to AUD has been studied focusing mostly on cognitive control (such as response inhibition, response selection, flexible updating), decision making, and reward learning (such as reward valuation, discount probability). Reduced cognitive flexibility and top–down, inhibitory control, linked to dysfunction in frontal-subcortical brain circuits, seems to confer vulnerability to AUD (63–65), and attentional bias to reward (66) and maladaptive reward learning are suggested to predict alcohol use progression (67). A longitudinal study with a 40-year follow-up, identified reduced cognitive efficiency as one of the two main premorbid predictors of both alcohol dependence and failure of remission later in life (68). Moreover, existing studies show an association between increased risk of AUD and low scores in broad intelligence tests and in cognitive functions encompassing attention and working memory (69), even though conflicting results are present in the literature (70,71). To this regard, controlled preclinical experiments could be useful to test which cognitive domains are associated with increased risk of AUD, without the confounding factors typical of human studies. [...]”*

1.2 Given the msP rats are alcohol-naïve, some of the comparisons of results to sleep changes reported in individuals with AUD are not entirely applicable.

We compared our results with individuals at risk of and with AUD because we believe that this translational approach might point towards alterations that are not the consequence of alcohol drinking, but rather endophenotypes which can shed light on the predisposing factors and mechanisms of early AUD development.

1.3 More information is expected to describe data acquisition software. Also, please make it clear whether data were individually hand scored or autoscored in batches. Autoscoring typically lacks reliability when compared to hand score

Data were individually hand scored We added more details about EEG/EMG acquisition and scoring in the Methods section which now reads as follow:

*“[...] Subsequently, rats were moved to the recording chamber, connected to a Pre-Amplifier (Intan Technologies, RHD 16-Channels) and to the Open Ephys acquisition board (30) and allowed to habituate for 48 hours before recording 24 hours of baseline. EEG and EMG signals we sampled at 1Khz using the open access Open Ephys Acquisition Software (GUI) and filtered (EEG: high-pass filter at 0.1 Hz; low-pass filter at 40 Hz; EMG: high-pass filter at 10 Hz; low-pass filter at 70 Hz). All data were down sampled at 512 Hz for off-line manual scoring (Sleep Sign, Kissei Comtec) of individual rats which occurred in 4s epochs following standard criteria (31). […]”*

30. Siegle JH, Cuevas López A, Patel YA, Abramov K, Ohayon S, Voigts J (2017) Open Ephys: an open-source, plugin-based platform for multichannel electrophysiology. *J Neural Eng* **14**: 045003

31. C. Iber, S. Ancoli-Israel, A. Cheson, *et al.* The AASM manual for the scoring of sleep and associated events: rules, terminology, and technical specifications (1st ed.), American Academy of Sleep Medicine, Illinois (2007)

1.4. Age of rats at time of recordings spans adolescence to adult? (average 58+/- 8 to 12 days). The range of age here is potentially problematic. Can data be separated and analyzed by age (although there may not a large enough group size for this and thus, additional data points for either age may be required). Sleep maturation during adolescence is an important factor to consider as sleep patterns undergo discernible changes including circadian rhythm shifts that could confound the early/late sleep measures here amongst other measures.

We thank the reviewer for highlighting this point and prompt us to elaborate further on it. The authors are aware that sleep and its electrophysiological features change during development and that, for instance, SWA declines across adolescence, especially between postnatal day (P) 20 and P60 in mice (de Vivo et al 2014) and P35 and P45 in rats (Olini et al 2013). Our goal was not to characterize sleep changes across development in the two rat lines, but to capture overall differences in sleep between late adolescent/young adult rats with different genetic backgrounds. This is why the two groups were balanced for age (they have the same average age of 58 days), so that, even if different ages are present within each group, across groups there is no significant difference (unpaired t-test p>0.9999). We performed a normality test (Shapiro-Wilk) which showed that both distributions are normal (Wistar p=0.3678, msP p=0.471), and a Grubbs test, which identified no outliers (Wistar G=1.619, msP G=1.98). We added this detailed analysis in the method section, under the Polysomnographic recordings paragraph. Moreover, we included a supplementary figure (S1) showing the age distribution in both groups (A) and the correlation between age and average SWA in the 24 hours for Wistar and msP (B). From the data depicted in the figure, it is clear that average SWA in the 24hours is quite stable throughout the age interval we chose, showing only a slight and non-significant decline in both groups (correlation msP R^2^=0.26 , p= 0.159, Wistar R^2^=0.023 , p=0.69).

de Vivo L, Faraguna U, Nelson AB, Pfister-Genskow M, Klapperich ME, Tononi G, Cirelli C. Developmental patterns of sleep slow wave activity and synaptic density in adolescent mice. Sleep. 2014 Apr 1;37(4):689-700, 700A-700B. doi: 10.5665/sleep.3570.

Olini N, Kurth S, Huber R. The effects of caffeine on sleep and maturational markers in the rat. PLoS One. 2013 Sep 4;8(9):e72539. doi: 10.1371/journal.pone.0072539. PMID: 24023748; PMCID: PMC3762801.

**Figure S1. Age and slow wave activity. A)** age at baseline recordings for each individual rat. W = Wistars, 58 ± 8 days, msP 58 ± 12 days, unpaired t-test p>0.99. **B)** mean NREM SWA levels (24 hours) across age from frontal cortex. Each symbol refers to a single animal.

1.5. Why were sleep spindles only assessed during “early and late sleep,” or first and last 3 hours of the light period? what was happening in the other 6 hours? Why are these period of interest more than peak rest time?

Sleep spindles are regulated by homeostatic and circadian processes (Borbély 1982) and their density, amplitude, and duration increase over successive NREMS cycles in humans (Dijk et al., 1993). The analysis of individual spindles, as well as slow waves, was focused on intervals of 3 hours at the beginning of sleep, when sleep pressure is high, and at the end of the sleep period, when sleep pressure is low, to capture the net change in these features over the course of sleep, and to more easily compare not only 24 hours average values between Wistar and msP, but also individual oscillation features in conditions of high and low sleep pressure within and across each rat line. The choice of the 3-hour interval is quite arbitrary but anyhow based on previous work by Vyazovskiy et al., 2007.

Borbély AA. A two process model of sleep regulation. ***Hum Neurobiol*** 1: 195–204, 1982.

Dijk DJ, Hayes B, Czeisler CA. Dynamics of electroencephalographic sleep spindles and slow wave activity in men: effect of sleep deprivation. Brain Res. 1993 Oct 29;626(1-2):190-9. doi: 10.1016/0006-8993(93)90579-c. PMID: 8281430.

Vyazovskiy VV, Riedner BA, Cirelli C, Tononi G. Sleep homeostasis and cortical synchronization: II. A local field potential study of sleep slow waves in the rat. Sleep. 2007 Dec;30(12):1631-42. doi: 10.1093/sleep/30.12.1631.

We added the proper reference in the results and we modified the text as follows (page 12): “*Since SWA merges different aspects of slow waves into a single metrics, we applied a slow wave detection algorithm to quantify multiple parameters of individual waves. To capture the net change of these features over the course of sleep, we focused the analysis on early (first 3 hours of light period) and late (last 3 hours of light period) NREM sleep (Fig 4D), corresponding to periods of high and low sleep pressure, respectively (35).”*

We have also computed the features of sleep spindles for the other 6 hours and prepared a supplementary figure S2 that shows that all spindle features (incidence, frequency, amplitude, duration, and number of oscillations) were reduced in msP rats relative to Wistar rats (unpaired t-test: incidence, t-test p=0.0009; frequency, t-test p=0.0001; amplitude, p<0.0001; duration p=0.0013; number of oscillations/spindle, t-test p=0.003).

**Fig. S2. Average spindle features during 6hr interval between zeitgeber time 4-9. A)** spindle incidence **B)** spindle amplitude. **C)** spindle duration. **D)** spindle frequency. **E)** number of oscillations per spindle. W=Wistar, unpaired t-test. ∗∗ p<0.01, ∗∗∗p<0.001.

Minor points for clarification:

1.6. If band pass on EEG was 0.1- 40 hz, why was beta power limited to 25 Hz?

We thank the reviewer for this comment. We clarify that we analysed a frequency range (15-25Hz) centred within the broader beta range (15-30Hz) as done by others (Leventhal et al 2012), however we have now computed the power within the interval 15-30Hz range and found equivalent results. We have now updated Figs.2 and 3 and modified the text accordingly.

Leventhal DK, Gage GJ, Schmidt R, Pettibone JR, Case AC, Berke JD. Basal ganglia beta oscillations accompany cue utilization. Neuron. 2012 Feb 9;73(3):523-36. doi: 10.1016/j.neuron.2011.11.032.

1.7. For post hoc analyses, why fisher LSD in some cases and Sidak’s MCT in others?

Fisher’s LSD test (without correction for multiple comparisons) was selected when our analysis focused on a limited number of scientifically meaningful comparisons designed to answer a single key question, specifically, whether a given physiological state or transition differed between Wistar and msP rats. We did not conduct exhaustive comparisons between physiological states themselves (e.g., NREM vs. REM or vs Wake), as these differences were not relevant to our hypotheses. For example, in updated Fig. 1C, our goal was to identify differences between msP and W within each physiological state, rather than between NREM in the light and dark phases. Since these states are inherently distinct, correction for multiple comparisons was unnecessary. Fisher’s LSD test was well-suited for detecting subtle group-specific differences in this context, offering high sensitivity when the number of comparisons is limited. This approach ensured a focused hypothesis-driven analysis while maintaining statistical rigor.

Conversely, Sidak’s multiple comparison test was used to analyse slow wave and spindle properties across early and late sleep stages and between rat lines. This analysis required a greater number of comparisons to address multiple scientific questions, such as whether early sleep spindles differed from late spindles within Wistar rats, within msP rats, or between the two groups. In this case, correction for multiple comparisons was necessary to maintain control over the family-wise error rate, ensuring robust statistical reliability. We have now included a Supplementary S1 Table with detailed results of post hoc tests and multiple comparisons.

1.8. Figure 1- “W” sometimes indicates Wistar and other times abbreviates “wake/waking,” please correct. For Figure 1A, figure says WAKE was n.s. but results text indicates significance- which is it? Statistical results are not detailed in Results for Figure 1C.

We thank the reviewer for these observations. Fig 1 has been updated adding representative hypnograms in 1A (as for request of reviewer 2), the significance in updated 1B wake, and spelling out wake in the graph axis. We added the statistical description for Fig.1C (now 1D in updated Fig1) in the Results which now read as follows:

“*A more detailed analysis showed that msP rats spend more time awake in the 2 first and last hours of the dark phase as well as in the first 2 hours of the light phase (Fig 1D, two-way repeated-measure ANOVA, wake: interaction time x rat line F(11, 176) = 1.903, p=0.041, rat line F(1, 16) = 4.37 p=0.053. NREM sleep: rat line F(1, 16) = 5.2, p=0.037, interaction time x rat line F(11, 176) = 1.4, p=0.17. REM sleep: interaction time x rat line F(11, 176) = 2.86 p=0.0018, rat line p=0.8, uncorrected Fisher’s LSD post hoc test), although this effect disappears after correcting for multiple comparisons*.*”*

In updated Fig.1F, we added a graph on the sleep fragmentation index, as suggested by reviewer #2, point 2.7, replacing the sleep onset graph.

In updated Fig.1H, we removed the statistical significance on the Wake-NREM sleep transition since the interaction between rat line and transition type was not significant and only a main effect of rat line is present in the ANOVA *rat line* F(1, 96) = 4.31 p=0.04.

Finally, we prepared supplementary S1Table for the detailed results of all post hoc comparisons performed in Fig.1I.

**Fig 1. Sleep architecture in Wistar and msP.** (A) Representative hypnograms for Wistar and msP rats. (B-C) Distribution of wake, NREM sleep, and REM sleep amount (mean ± std) in the 24h (B) and during light and dark periods (C). (D) Twenty-four-hour time course of time spent in each state (mean ± SEM). Grey areas indicate the dark cycle, from 8:00pm to 8:00am. (E) Distribution of the number of episodes in the light, L, and dark, D, phase (mean ± std). (F) Number of sleep attempts during the 24 hours. (G) Sleep fragmentation index computed as the number of wakefulness episodes / total sleep time. (H) Number of transitions between stages. (I) Percentage of time spent in active or quiet wake during the light, L, and dark, D, period. Empty circles represent individual rats (Wistar n=9, msP n=9). * p<0.05, ** p<0.01.

1.9. In results, the time is referred to as a percentage, but the graphs are showing time in minutes, please make them match.

We added the time in minutes in the Results paragraph:

“Across the 24 hours, msP rats spent 7.5% more time awake and less time in NREM sleep relative to Wistar rats (Fig. 1A-C; msP: 726.6 ± 44.27 minutes in wake, 566.2 ± 42.19 in NREM sleep, 145.9 ±37.04 in REM sleep; Wistars: 683.8 ± 42.58 minutes in wake, 612.3 ± 43.63 in NREM sleep, 143.8 ± 20.63 minutes in REM sleep; 2-way ANOVA, interaction *stage* x *rat line* F(2, 48) = 5.793, p=0.0056, Fisher’s LSD post-hoc test msP vs Wistar wake p=0.0247, NREM sleep p=0.0163, Fig 1A; 2-way ANOVA, *stage* x *rat line* F(5, 96)=3.9 p=0.0029, NREM dark phase p=0.014, Wake p=0.0014, Fig 1C).”

1.10. Figure 4B, does not appear to be “change in uV2.”

We modified the axis legend for all power spectra to µV^2^/0.25Hz.

**Reviewer #2:** The manuscript by de Vivo et al. characterized the sleep phenotype of a preclinical rat model of patients with AUD. Overall this investigation was conducted adequately providing a baseline dataset for this specific rat model which, in future experiments, will be useful to deeper understanding the neural circuits at the basis of substance use vulnerability.

We thank the reviewer for the overall positive comment.

However, I have some major methodological and conceptual concerns:

2.1. I believe this work would have been more complete if the authors had included groups exposed to alcohol (possibly using a longitudinal protocol). Limiting the sleep profiles of this model to baseline conditions is of course of interest, however, it would have been nice to see what specific sleep changes are due to alcohol exposure rather than genetic predisposition. Please comment on this.

We appreciate the reviewer's comment and acknowledge that the lack of alcohol exposure is a limitation of our study, as noted at the end of the discussion (and also by Reviewer #1, point 1.2). However, the effects of acute alcohol exposure on sleep have been extensively studied in both humans and animal models (Abrahao et al., 2020; Colrain et al., 2014; Chan et al, 2013; Thakkar et al., 2015), whereas research on sleep microarchitecture and global brain activity in substance-naive individuals and animal models at increased risk for AUD remains limited. This gap in knowledge is of particular interest to us. We believe that examining inherent sleep disturbances and global brain activity alterations in individuals predisposed to AUD, before alcohol consumption, can provide valuable insights into the predisposing factors and mechanisms underlying AUD development.

Nevertheless, we have expanded the discussion clearly stating the limitations present in our study (see point 2.2) and we have added references to studies on the effects of acute alcohol exposure on sleep in the introduction section.

Abrahao KP, Pava MJ, Lovinger DM. Dose-dependent alcohol effects on electroencephalogram: Sedation/anesthesia is qualitatively distinct from sleep. Neuropharmacology. 1 March 2020;164:107913.

Colrain IM, Nicholas CL, Baker FC. Chapter 24 - Alcohol and the sleeping brain. In: Sullivan EV, Pfefferbaum A, curatori. Handbook of Clinical Neurology. Elsevier; 2014. p. 415–31. (Alcohol and the Nervous System; vol. 125). https://www.sciencedirect.com/science/article/pii/B9780444626196000240

Chan JK, Trinder J, Andrewes HE, Colrain IM, Nicholas CL. The acute effects of alcohol on sleep architecture in late adolescence. Alcohol Clin Exp Res. 2013 Oct;37(10):1720-8. doi: 10.1111/acer.12141. Epub 2013 Jun 25.

Thakkar MM, Sharma R, Sahota P. Alcohol disrupts sleep homeostasis. Alcohol. June 2015;49(4):299–310.

2.2. Similarly, the authors claim that the altered sleep profile in msP rats might entail memory consolidation and behavioral impairments. However, since they did not provide any data on these aspects, I suggest reporting these speculations in a specific limitation paragraph together with the reply of my previous point.

We thank the reviewer for this comment, and we now include a paragraph on the limitations of our study:

*“[...] This study has several limitations, including the lack of alcohol exposure to measure the direct association between sleep EEG features and the amount or pattern of voluntary alcohol drinking, as well as the effects of ethanol on sleep microarchitecture in msP rats. However, extensive research has characterized the effects of acute alcohol consumption on sleep and EEG activity in both individuals with AUD and healthy controls (reviewed in (5,70)). Additionally, one study found that binge alcohol treatment affects sleep-wake activity differently in alcohol-preferring (P) and non-preferring (NP) rats, particularly during the dark period following ethanol exposure (71). P rats were more susceptible to alcohol, exhibiting a significant reduction in NREM sleep, increased wakefulness, and no change in REM sleep compared to NP rats.*

*Similarly, the current study would have benefited from in vitro electrophysiological recordings and behavioral tests linking the blunted SW homeostasis and reduced spindle activity in msP rats with specific deficits in synaptic plasticity, learning, and memory consolidation. […]*

2.3. I noticed the presence of outliers in several figures, and I feel that, at least some of them, importantly impacted on the statistics and the conclusion of the present work. In particular, fig. 1F (msp group), 1G (W-NREM, wistar group), 4C (msp group), 5B (w group). Did the authors check for the weight of them on their conclusions? Did they apply any automatic outlier detection?

We thank the reviewer for this relevant comment. We decided not to remove data points from the analysis to provide the most realistic representation of the results. However, we are sensitive to reviewer comment, therefore have performed Grubbs test for outliers detection in all datasets to check their weight on the results and found that:

- Figure 1F, now 1E, (sleep attempts in 24 hr), no outlier was identified.

| Grubbs (Alpha = 0.05) | W: G = 2,209 | msp: G = 2,134 |
| --- | --- | --- |

- Figure 1G, now 1H (transitions Wake-NREM, Wistar group): one outlier was identified

| Grubbs (Alpha = 0.05) | W: G = 2,493 | msp: G = 1,421 |
| --- | --- | --- |

Notably, removing this data point from the analysis resulted in no significant effect of rat line in the ANOVA F(1, 95)=3.26 p=0.074; interaction state x rat line F(5,95)=0.99 p=0.43, transition F(5,96)=246.2 p<0.0001.

- Fig.1H, now 1I. One outlier was found in Wistar, Active wake D. Removing this data point did not significantly change the results. Two-way ANOVA rat line F(1,63)=0.13 p=0.7, interaction rat line x state F(3.63)=6.07 p=0.0011, post hoc Wistar vs msP in Active wake L p=0.016, in Quiet wake L p= 0.0164.
- Figure 4B, one outlier was identified in msP. Removing it from analysis did not affect the result since Mann-Whitney test is still significant (p=0.0037 instead of 0.024).

- Figure 4C, msP group (dark period SWA change), there was no outlier, however, we corrected an error present in the previous version of the graph. Mann-Whitney test is still significant p=0.0244.
- Figure 4G, slow wave frequency, one outlier was identified in msP early sleep. Removing this data point resulted in a significant difference between msP and Wistars in late sleep, instead of a trend (repeated measure 2-way ANOVA, time F(1,15)=3.56 p=0.078, rat line F(1,16)=2.55 p=0.13, interaction rat line x time F(1,15)=4.98 p=0.041, Sidak’s multiple comparison test msP vs Wistar late sleep p=0.035; in Wistars, early vs late sleep p=0.0185). Below, we report SW frequency with all data points (left) and without the outlier (right).

- Figure 4H, slow wave duration, one outlier was identified in msP early sleep. Removing this data point did not change the main results of the analysis (repeated measure 2-way ANOVA, time F(1,15)=6.13 p=0.026, rat line F(1,16)=5.39, p=0.034, interaction rat line x time F(1.15)=6.37 p=0.023, Sidak’s multiple comparison test msP vs Wistar in late sleep p=0.0046; in Wistars, early vs late sleep p=0.0051). Below, we report both graphs with (left) and without (right) outlier.

We added to Fig.4 the statistics for the early vs late sleep comparisons, and we updated the order of the graphs and the text in the result session to improve the description of all the differences, as follows:

“*[...] In late sleep, slow waves had shorter duration in msP relative to Wistars (Fig. 4G, repeated measure 2-way ANOVA interaction time x rat line F (1, 16) = 10.64 p=0.005, Sidak’s multiple comparison test late sleep, msP vs Wistar p=0.018, early vs late sleep Wistar p=0.0047, msP 0.55). Moreover, slow waves had faster frequency (Fig.4H, repeated measure, 2-way ANOVA interaction rat line x time F(1,16)=9.65 p=0.0068 Sidak’s multiple comparison test early vs late sleep, Wistar p=0.0128, msP p=0.4) and were more numerous in early than in late sleep in Wistar rats but not in msP (Fig.4I, repeated measure 2-way ANOVA, interaction time x rat line F (1, 16) = 13.55 p=0.002, Sidak’s multiple comparison test early vs late sleep Wistar p<0.0001, msP p=0.44)*.”

Updated Fig.4 is reported below:

**Fig 4. SWA and sleep slow waves.** (A) Twenty-four-hour time course of SWA (mean±SEM), two-way ANOVA interaction rat line x time p<0.0001, uncorrected Fisher LSD post hoc test p=0.02. The grey area indicates 12hrs of lights off. (B) SWA change during the light period (1^st^ hour – 12^th^ hour of light period, Mann-Whitney test p=0.024), mean±std. (C) SWA during the dark period (1^st^ hour – 12^th^ hour of dark period, Mann-Whitney test p=0.0244), mean±std. (D) Representative EEG signal (band-pass filtered 0.5-4Hz) during early and late sleep for Wistar (upper part) and msP (lower part). (E) Values of slow wave amplitude during early and late sleep. (F) Slope of slow waves during early and late sleep. (G) Duration of slow waves. (H) Frequency of slow waves during early and late sleep. (I) Incidence of slow waves. Columns indicate mean ± SEM. Empty circles are single rats. Data in E-I were analyzed with repeated measures 2-way ANOVA followed by Sidak’s multiple comparison test, see Supplementary Table 1 for detailed statistical results. * p<0.05, ** p<0.01, **** p<0.0001.

- Figure 5B, sigma increase during the light phase, one outlier was identified in the Wistar group (W G=2.263, msP G=1.882). Removing this outlier from the analysis did not change the result, the Mann-Whitney test remained significant, p=0.011. Below, we report the graph with (left) and without (right) the outlier.

We added to Fig. 5 the comparisons between early and late sleep. Exact p values for all post hoc comparisons have been added to S1 Table, and graphs without the outliers have been added as Figure S3 in the supporting information.

Based on the fact that the presence of these outliers does not substantially modify the statistical results, we would prefer not to exclude them to provide a full representation of the results. In this revised version of the manuscript, we now provide the graphs and analysis of the data without the outliers in Fig.S3. However, if the reviewer considers the exclusion of the outliers mandatory, we will be happy to revise the manuscript accordingly.

The only case in which removing the outlier resulted in a loss of significance was in the number of transitions from wake to NREM sleep (updated Fig.1H). However, we used this result to support the claim that msP rats have a more fragmented wake-sleep pattern, which is also confirmed by the higher number of waking and sleep episodes (updated Fig. 1E; two-way ANOVA, *rat line* F(1,90) = 4.68 p=0.033), the higher number of sleep attempts across the 24 hours (updated Fig 1F), and the higher sleep fragmentation index (updated Fig.1G). Therefore, we believe that this claim is real and not due to the presence of outliers. Nevertheless, we include the results without the outliers in Fig. S3.

2.4. How did the authors correct for multiple comparisons in fig. 1c?

We did not correct for multiple comparisons in Fig.1C, we used the Fisher LSD test. Correcting for multiple comparisons resulted in non significant post hoc tests, which is now clearly stated in the main result session:

“[*...] A more detailed analysis showed that msP rats spend more time awake in the 2 first and last hours of the dark phase as well as in the first 2 hours of the light phase (Fig 1C, two-way repeated-measure ANOVA, wake: interaction time x rat line F(11, 176) = 1.903, p=0.041, rat line F(1, 16) = 4.37 p=0.053. NREM sleep: rat line F(1, 16) = 5.2, p=0.037, interaction time x rat line F(11, 176) = 1.4, p=0.17. REM sleep: interaction time x rat line F(11, 176) = 2.86 p=0.0018, rat line p=0.8, uncorrected Fisher’s LSD post hoc test), although this effect disappears after correcting for multiple comparisons*.”

Two way repeated measure ANOVA, time x rat line, Fisher LSD test.

| **stage** | **Anova factor** | **F** | **p value** | **Fisher LSD test.** |
| --- | --- | --- | --- | --- |
| Wake | time x rat line | F(11,176)=1.903 | 0.0417 | 2 hr p=0.011 14hr p=0.0134  24hr p=0.0298 |
|  | rat line | F(1,16)=4.37 | 0.053 |  |
| NREM sleep | time x rat | F(11,176)=1.402 | 0.175 |  |
|  | rat line | F(1,16)=5.19 | 0.037 | 2 hr p=0.0088 14hr p=0.0459 24hr p=0.0226 |
| REM sleep | time x rat line | F(11,176)=2.85 | 0.0018 | 14 hr p=0.0045  16hr p=0.0286 |
|  | rat line | F(1,13)=0.022 | 0.88 |  |

2.5. Fig. 1D, the statistics in the text seems referring to a main ANOVA group effect rather than an interaction effect before applying the corrected post-hoc analysis.

We thank the reviewer for this comment. We removed the post-hoc analysis from Fig. 1D since the interaction between rat line and state is not significant.

Effect of rat line p=0.0331, F (1, 90) = 4.681

effect of state p<0,0001; F (5, 90) = 126.9
Interaction p=0.3514 , F (5, 90) = 1.128

I also have some minor concerns/suggestions:

2.6. For figures 4E-4I, 5D-H, how did the authors choose to limit the SWA and spindle analyses to the first and last 3 hours of NREM sleep? Could you provide any reference for this choice?

We chose to focus our analysis of SWA and spindle features to the beginning and the end of the light period, corresponding to times of high and low sleep pressure, respectively (see also answer to reviewer #1, point 1.5).

The specific interval of 3 hours is rather subjective but anyhow based on a seminal paper on sleep homeostasis: Vyazovskiy VV, Riedner BA, Cirelli C, Tononi G. Sleep homeostasis and cortical synchronization: II. A local field potential study of sleep slow waves in the rat. Sleep. 2007 Dec;30(12):1631-42. doi: 10.1093/sleep/30.12.1631.

We added this reference to the paper.

2.7. I would suggest including the sleep fragmentation index (n° of wakefulness episodes / total sleep time) to make easier (for the readers) the conclusions on this characteristic

We are thankful for this suggestion. We computed the sleep fragmentation index and found that it is higher in msP relative to Wistars. We added this result to Fig.1G and in the main text.

Wistar: 33.47 ± 7.123

msp: 40.36 ± 5.62

unpaired t-test p=0.0366

grubbs test no outlier

| (Alpha = 0.05) | G = 2,146 | G = 1,565 |
| --- | --- | --- |

2.8 I would also suggest including representative hypnograms to readily appreciate the different sleep structure between groups.

As suggested by the reviewer, w have added representative hypnograms for Wistar and msP rats to Fig1.
