# Supplementary material for: Sleep phenotyping in a rat model of susceptibility to substance use disorders
Source: PLoS One. 2025 May 29;20(5):e0324459. doi: 10.1371/journal.pone.0324459 (PMC12121824; doi:10.1371/journal.pone.0324459)
Supplement: Attachment — Submitted filename: Response to Reviewers.docx [file pone.0324459.s006.docx]

Reviewer #1: The authors have adequately addressed this reviewer's concerns, in large part, and the manuscript is much improved from the previous version.

We thank the reviewer for constructive suggestions and for acknowledging our efforts.

It is not clear, however, why the authors chose to present data both with (main figures) and without (supplemental figure S3) outliers. This reviewer would prefer the authors present only the figures and statistics that exclude outliers in the main body, and possibly only include figures with outliers as a supplement if they feel it necessary to show both. Reporting a statistically significant result that hinges on an outlier does little to enhance the reader's interpretation of the data.

We are now presenting figures and statistics excluding all identified outliers in the main body.

Reviewer #2: I have no furhter comments and I agree with the authors that the outlier analyses can be kept in the supplementary material.

Thank you.

Editor’s comment: Specifically, I agree with Reviewer 1 that statistical issues were not adequately addressed in the first revision of your manuscript.

From the information that is available to us, reviewer #1 did not raise further concerns regarding the statistical analysis we carried out in the first revision. He/she asked to provide in the main body the results excluding the outliers, which is what we are implementing in this second revision.

Moreover, the Grubbs test is designed to identify single outliers from relatively normal distributions, which may have not been always the case in your dataset, and ANOVA is sensitive to non-normal distributions as well as to outliers.

We extensively examined our dataset to detect deviations from normality through Shapiro-Wilk tests and the examination of residuals and Q-Q plots. This analysis showed that in most cases our data followed a normal distribution which allowed us to apply parametric statistics such as ANOVAs and t-tests. The few exceptions identified, in which data significantly deviated from normality, have been addressed by applying non-parametric tests.

Regarding the outlier analysis, we have now systematically checked for outliers in our entire dataset, applying both Grubbs’ test with alfa=0.05 and ROUT test with maximum False Discovery Rate (Q) of 1%. ROUT test is designed to identify more than one outlier (Motulsky et al., 2006) even though Grubbs’ test is more efficient in identifying single outliers. The two methods gave almost identical results.

We now provide a detailed description of all tests applied in Supplementary Table 1 and provide results excluding the outliers identified with the Grubbs’ test in the main body, as requested by reviewer #1.

Motulsky, H.J., Brown, R.E. Detecting outliers when fitting data with nonlinear regression – a new method based on robust nonlinear regression and the false discovery rate. *BMC Bioinformatics* **7**, 123 (2006). https://doi.org/10.1186/1471-2105-7-123

On the other hand, your results seem quite robust to different statistical approaches, and I understand your choice to provide a full representation of your results in the main manuscript.

On these bases, I recommend that you perform a further minor revision of the analysis and presentation of your results to fully address these issues, possibly seeking the advice of a professional biostatistician. You may consider consistently using non-parametric tests instead of parametric tests to decrease the sensitivity of results to potential outliers.

Since our data are mainly normally distributed, parametric statistics seem appropriate. The few exceptions where normality was violated were addressed with the use of non-parametric tests. We decided to remove outliers, as suggested by reviewer#1, based on the Grubbs’ test, since the ROUT test gave identical results.

Please provide a detailed description of your statistical approach, including the identification and management of outliers, in the methods section of the main manuscript, and discuss any related limitation in the discussion section.

We included the description of all outliers identified and statistical approaches in Supplementary table 1 and modified the method section as follows:

*[…] Normality of data was evaluated using the Shapiro–Wilk test, alongside inspection of QQ plots and residuals. For datasets that violated normality assumptions, nonparametric tests were used; otherwise, parametric tests were conducted. The presence of outliers was tested using Grubbs’ test (alpha=0.05), most sensitive to single outliers, and ROUT test (False Discovery Rate Q=1%)), which applies non-linear regression to flag multiple outliers while controlling for false discovery rate (Motulsky et al., 2006). When outliers were identified, they were excluded from subsequent analyses. In case of missing values, data were analyzed by fitting mixed-effects models. Supplementary Table S1 provides a summary of outliers found and the statistical tests applied.*
